# Supplementary material for: Validity and Reliability of a Questionnaire on Attitudes, Knowledge, and Perceptions of Pharmacy Students Regarding the Training Received on Antibiotics and Antimicrobial Resistance during Their University Studies
Source: Antibiotics (Basel). 2024 Aug 26;13(9):811. doi: 10.3390/antibiotics13090811 (PMC11428262; doi:10.3390/antibiotics13090811)
Supplement: Supplementary file 1 [file antibiotics-13-00811-s001.zip › antibiotics-3104815-supplementary file S1.pdf]

CUESTIONARIO FACULTAD DE FARMACIA

El siguiente cuestionario está dirigido a estudiantes de farmacia.
Por favor, conteste las siguientes preguntas con sinceridad. No le llevará más de 10 minutos.

El propósito de este estudio es aprender cómo los estudiantes de farmacia en Europa son formados sobre los antibióticos y su uso. Agradecemos mucho que complete el cuestionario. Los resultados serán utilizados para sugerir mejoras en los planes de estudio vigentes y serán publicados en revistas revisadas por pares de nivel nacional e internacional. El cuestionario requiere entre cinco y diez minutos para ser completado. Por favor, intente responder a todas las preguntas. Todas las respuestas son anónimas y serán almacenadas confidencialmente.

Ítem 1: ¿Qué edad tiene (en años)?

Ítem 2: ¿Cuál es su sexo?

- o Masculino
- o Femenino

Ítem 3: ¿Es usted ciudadano del país en el que estudia farmacia?

- o Sí
- o No

Ítem 4: ¿Cuál es la duración total de su formación farmacéutica en su facultad?

- o 4 años
- o 5 años
- o 5,5 años/11 semestres
- o 6 años
- o 7 años
- o Otros

Ítem 5: ¿En qué facultad de farmacia estudia?

- o USC
- o Otros

Ítem 6: Si otros, ¿cuál? (nombre de la facultad)

Para cada pregunta, responda “me siento… (0=nada preparado, 5=suficientemente preparado, 9=muy preparado) para…”
Si hay preguntas en las que no ha tenido ninguna formación, seleccione “no se recibió formación”, independientemente de que tan bien preparado se sienta sobre ese tema.

Dimensión 1:

Calidad del cuidado.

Me siento capacitado…

	0	1	2	3	4	5	6	7	8	9	No se recibió formación
**Ítem 7:** Para comprender la motivación del uso de antibióticos en el entorno clínico	○	○	○	○	○	○	○	○	○	○	○
**Ítem 8:** Para trabajar dentro del equipo multidisciplinar dedicado al manejo de los antibióticos en los hospitales	○	○	○	○	○	○	○	○	○	○	○

Dimensión 2:

Habilidades comunicativas.

Me siento capacitado…

	0	1	2	3	4	5	6	7	8	9	No se recibió formación

**Ítem 9:** Para comunicar al paciente las necesidades o limitaciones del uso de antibióticos en el entorno clínico	○	○	○	○	○	○	○	○	○	○	○
**Ítem 10:** Para comunicarme de manera efectiva dentro del equipo multidisciplinar dedicado al manejo de los antibióticos en los hospitales	○	○	○	○	○	○	○	○	○	○	○

<u>Dimensión 3:</u>

Resistencia antibiótica.

Me siento capacitado…

	0	1	2	3	4	5	6	7	8	9	No se recibió formación
**Ítem 11:** Para utilizar el conocimiento sobre los mecanismos comunes de resistencia a los antibióticos en los patógenos	○	○	○	○	○	○	○	○	○	○	○

**Ítem 12:** Para utilizar el conocimiento sobre la epidemiología de la resistencia bacteriana, incluyendo variaciones locales/regionales											
**Ítem 13:** Para practicar el control efectivo de la infección e higiene (para prevenir la diseminación de las bacterias)											
**Ítem 14:** Para usar el conocimiento sobre las consecuencias negativas del uso de los antibióticos (resistencia bacteriana, efectos tóxicos o adversos, coste, infecciones por *Clostridium difficile*)											

¿Cuáles de los siguientes métodos se han utilizado en su facultad para formar sobre el uso responsable de los antibióticos (los temas del anterior conjunto de preguntas) y cuán útiles fueron?

<u>Dimensión 4:</u>

Metodología de la facultad.

	No ha sido utilizado	No útil en absoluto	Neutral	Útil	Muy útil	Estoy inseguro	No he entendido la pregunta

**Ítem 15:** Conferencias con menos de 15 personas	○	○	○	○	○	○	○
**Ítem 16:** Enseñanza en grupos reducidos	○	○	○	○	○	○	○
**Ítem 17:** Discusión de casos clínicos y viñetas	○	○	○	○	○	○	○
**Ítem 18:** Tareas de aprendizaje activo (lectura de artículos, trabajo en grupo, preparación de presentación oral)	○	○	○	○	○	○	○
**Ítem 19:** Aprendizaje electrónico (e-learning)	○	○	○	○	○	○	○
**Ítem 20:** Role play o sesiones de habilidades comunicativas tratando con pacientes que demandan antibioterapia	○	○	○	○	○	○	○

**Ítem 21:** Rotación durante práctica clínica en unidades de enfermedades infecciosas (involucrando pacientes)	○	○	○	○	○	○	○
**Ítem 22:** Rotación en microbiología	○	○	○	○	○	○	○
**Ítem 23:** Enseñanza tutorizada o casi tutorizada (clase impartida por otros estudiantes o farmacéuticos recientemente graduados)	○	○	○	○	○	○	○

<u>Dimensión 5:</u>

Formación en antibióticos en la facultad.

Ítem 24: En general, ¿cree que ha recibido suficiente formación en la facultad de farmacia sobre el uso de antibióticos para su práctica futura?

- Sí
- No. Siento que tuve suficiente formación sobre el tratamiento antibiótico en general, pero necesito más formación sobre el uso responsable de antibióticos.
- No. Siento que necesito más formación tanto sobre el tratamiento antibiótico en general como sobre el uso prudente de antibióticos.
- No estoy seguro

Ítem 25: ¿Alguno de los exámenes de la facultad de farmacia ha incluido preguntas sobre el tratamiento con antibióticos?

- Sí
- No
- No estoy seguro
- Otros

Ítem 26: Otros, por favor especificar:

Ítem 27: ¿Cómo cree que se puede mejorar la formación sobre el tratamiento antibiótico y el uso prudente de antibióticos?

Ítem 28: ¿Cómo encontró el lenguaje utilizado en este cuestionario?

- Pude entender todo o casi todo
- Pude entender la mayoría de las preguntas
- Muchas preguntas me resultaron difíciles de entender
- La mayor parte del cuestionario me resultó difícil de entender

PHARMACY FACULTY QUESTIONNAIRE

The following questionnaire is directed at pharmacy students.

Please answer the following questions sincerely. It will take no more than 10 minutes.

The purpose of this study is to learn how pharmacy students in Europe are trained about antibiotics and their use. We greatly appreciate your completion of the questionnaire. The results will be used to suggest improvements in current curricula and will be published in nationally and internationally peer-reviewed journals. The questionnaire takes between five and ten minutes to complete. Please try to answer all questions. All responses are anonymous and will be stored confidentially.

Item 1: How old are you (in years)?

Item 2: What is your gender?

- o Male
- o Female

Item 3: Are you a citizen of the country where you are studying pharmacy?

- o Yes
- o No

Item 4: What is the total duration of your pharmacy training at your faculty?

- o 4 years
- o 5 years
- o 5,5 years/11 semesters
- o 6 years
- o 7 years
- o Other

Item 5: Which pharmacy faculty are you studying at?

- o USC
- o Otros

Item 6: If other, which one? (name of the faculty)

For each question, answer "I feel… (0=not prepared at all, 5=adequately prepared, 9=very

prepared) to…" If there are questions in which you have not received any training, select "no training received," regardless of how well-prepared you feel about that topic.

Dimension 1:

Quality of care.

I feel qualified…

	0	1	2	3	4	5	6	7	8	9	No training received
**Item 7:** To understand the motivation for antibiotic use in the clinical setting	○	○	○	○	○	○	○	○	○	○	○
**Item 8:** To work within the multidisciplinary team dedicated to the management of antibiotics in hospitals	○	○	○	○	○	○	○	○	○	○	○

Dimension 2:

Communicative skills.

I feel qualified…

	0	1	2	3	4	5	6	7	8	9	No training received

**Item 9:** To communicate to the patient the needs or limitations of antibiotic use in the clinical setting	◯	◯	◯	◯	◯	◯	◯	◯	◯	◯	◯
**Item 10:** To communicate effectively within the multidisciplinary team dedicated to the management of antibiotics in hospitals	◯	◯	◯	◯	◯	◯	◯	◯	◯	◯	◯

Dimension 3:

Antibiotic Resistance.

I feel qualified…

	0	1	2	3	4	5	6	7	8	9	No training received
**Item 11:** To use knowledge about common antibiotic resistance mechanisms in pathogens	◯	◯	◯	◯	◯	◯	◯	◯	◯	◯	◯
**Item 12:** To use knowledge about the epidemiology of bacterial resistance, including local/regional variations	◯	◯	◯	◯	◯	◯	◯	◯	◯	◯	◯

**Item 13:** To practice effective infection control and hygiene (to prevent the spread of bacteria)	○	○	○	○	○	○	○	○	○	○	○
**Item 14:** To use knowledge about the negative consequences of antibiotic use (bacterial resistance, toxic or adverse effects, cost, Clostridium difficile infections)	○	○	○	○	○	○	○	○	○	○	○

Which of the following methods have been used at your school to educate about the responsible use of antibiotics (the topics in the previous set of questions) and how useful were they?

<u>Dimension 4:</u>

Faculty methodology.

	Has not been used	Not useful at all	Neutral	Usef ul	Very useful	I'm insecure	I have not understood the question
**Item 15:** Conferences with less than 15 people	○	○	○	○	○	○	○
**Item 16:** Teaching in small groups	○	○	○	○	○	○	○

**Item 17:** Discussion of clinical cases and vignettes	◯	◯	◯	◯	◯	◯	◯
**Item 18:** Active learning tasks (reading articles, group work, oral presentation preparation)	◯	◯	◯	◯	◯	◯	◯
**Item 19:** Electronic learning (e-learning)	◯	◯	◯	◯	◯	◯	◯
**Item 20:** Role play or communication skills sessions dealing with patients who demand antibiotic therapy	◯	◯	◯	◯	◯	◯	◯
**Item 21:** Rotation during clinical practice in infectious disease units (involving patients)	◯	◯	◯	◯	◯	◯	◯
**Item 22:** Rotation in microbiology	◯	◯	◯	◯	◯	◯	◯

**Item 23:** Tutored or quasi-tutored teaching (class taught by other students or recently graduated pharmacists)	○	○	○	○	○	○	○

Dimension 5:

Training in antibiotics at the faculty.

Item 24: Overall, do you think you have received enough training in pharmacy school on antibiotic use for your future practice?

- o Yes
- o No. I feel like I had enough training on antibiotic treatment in general, but I need more training on responsible antibiotic use.
- o No. I feel that I need more training both on antibiotic treatment in general and on the prudent use of antibiotics.
- o I'm not sure

Item 25: Have any of your pharmacy school exams included questions about antibiotic treatment?

- o Yes
- o No
- o I'm not sure
- o Other

Item 26: Others, please specify:

Item 27: How do you think training on antibiotic treatment and the prudent use of antibiotics can be improved?

Item 28: How did you find the language used in this questionnaire?

- I could understand everything or almost everything
- I was able to understand most of the questions
- Many questions were difficult for me to understand.
- I found most of the questionnaire difficult to understand.
