# Supplementary material for: Validity and Reliability of a Questionnaire on Attitudes, Knowledge, and Perceptions of Pharmacy Students Regarding the Training Received on Antibiotics and Antimicrobial Resistance during Their University Studies
Source: Antibiotics (Basel). 2024 Aug 26;13(9):811. doi: 10.3390/antibiotics13090811 (PMC11428262; doi:10.3390/antibiotics13090811)
Supplement: Supplementary file 1 [file antibiotics-13-00811-s001.zip › antibiotics-3104815-supplementary.pdf]

Table S1. Comparison of total variances explained in different PCAs.

Total variance explained											
**PCA of original data**				**Ridge PCA**				**Bootstrapping PCA**			
**Component**	**Initial eigenvalues**			**Component**	**Initial eigenvalues**			**Component**	**Initial eigenvalues**		
	**Total**	**% Explained variance**	**% Cumulative variance**		**Total**	**% Explained variance**	**% Cumulative variance**		**Total**	**% Explained variance**	**% Cumulative variance**
**1**	**6,178**	**34,323**	**34,323**	**1**	**6,345**	**34,323**	**34,323**	**1**	**6,792**	**37,668**	**37,668**
**2**	**3,638**	**20,209**	**54,532**	**2**	**3,736**	**20,209**	**54,532**	**2**	**3,557**	**19,724**	**57,393**
**3**	**1,472**	**8,178**	**62,711**	**3**	**1,512**	**8,178**	**62,711**	**3**	**1,805**	**10,010**	**67,402**
**4**	**1,197**	**6,649**	**69,360**	**4**	**1,229**	**6,649**	**69,360**	**4**	**1,311**	**7,270**	**74,673**
**5**	**1,055**	**5,862**	**75,222**	**5**	**1,084**	**5,862**	**75,222**	**5**	**1,001**	**5,545**	**80,218**
6	,733	4,074	79,296	6	0,753	4,074	79,296	6	0,769	4,263	84,480
7	,558	3,101	82,396	7	0,573	3,101	82,396	7	0,616	3,414	87,894
8	,534	2,967	85,364	8	0,549	2,967	85,364	8	0,505	2,803	90,697
9	,458	2,544	87,907	9	0,470	2,544	87,907	9	0,413	2,292	92,989
10	,440	2,445	90,352	10	0,452	2,445	90,352	10	0,336	1,864	94,854
11	,410	2,278	92,630	11	0,421	2,278	92,630	11	0,268	1,488	96,342
12	,378	2,099	94,729	12	0,388	2,099	94,729	12	0,208	1,153	97,495
13	,269	1,495	96,223	13	0,276	1,495	96,223	13	0,158	0,875	98,370
14	,194	1,076	97,299	14	0,199	1,076	97,299	14	0,116	0,644	99,013
15	,185	1,028	98,327	15	0,190	1,028	98,327	15	0,082	0,456	99,470
16	,144	,800	99,127	16	0,148	,800	99,127	16	0,053	0,294	99,764
17	,110	,610	99,738	17	0,113	,610	99,738	17	0,030	0,165	99,929
18	,047	,262	100,000	18	0,048	0,262	100,000	18	0,013	0,071	100,000

Extraction method: Principal Component Analysis.

Table S2. Comparison of total variances explained in PCA original data and PCA cross-validation.

Total variance explained							
**PCA of original data**				**Cross validation PCA**			
**Component**	**Initial eigenvalues**			**Component**	**Initial eigenvalues**		
	**Total**	**% Explained variance**	**% Cumulative variance**		**Total**	**% Explained variance**	**% Cumulative variance**
**1**	**6,178**	**34,323**	**34,323**	**1**	**6,095**	**33,070**	**33,070**
**2**	**3,638**	**20,209**	**54,532**	**2**	**3,291**	**18,780**	**51,850**
**3**	**1,472**	**8,178**	**62,711**	**3**	**1,468**	**8,380**	**60,230**
**4**	**1,197**	**6,649**	**69,360**	**4**	**1,178**	**6,730**	**66,960**
**5**	**1,055**	**5,862**	**75,222**	**5**	**1,038**	**5,920**	**72,880**
6	,733	4,074	79,296	6	0,745	4,250	77,130
7	,558	3,101	82,396	7	0,608	3,470	80,600
8	,534	2,967	85,364	8	0,544	3,100	83,700
9	,458	2,544	87,907	9	0,497	2,840	86,540
10	,440	2,445	90,352	10	0,433	2,470	89,010
11	,410	2,278	92,630	11	0,380	2,160	91,170
12	,378	2,099	94,729	12	0,333	1,890	93,060
13	,269	1,495	96,223	13	0,285	1,620	94,680
14	,194	1,076	97,299	14	0,237	1,340	96,020
15	,185	1,028	98,327	15	0,195	1,100	97,120
16	,144	,800	99,127	16	0,156	0,880	98,000
17	,110	,610	99,738	17	0,120	0,680	98,680
18	,047	,262	100,000	18	0,095	0,530	99,210
				19	0,075	0,420	99,630
				20	0,050	0,370	100,000

Extraction method: Principal Component Analysis.
